# Corrosion Behavior of Biocompatible Ti3Mn Alloy in Different Physiological Conditions for Biomedical Applications

**DOI:** 10.3390/ma18184346

**Published:** 2025-09-17

**Authors:** Clara Mihaela Soare, Cristina Jimenez-Marcos, Santiago Brito-Garcia, Julia Claudia Mirza-Rosca, Ionelia Voiculescu

**Affiliations:** 1 Quality Engineering and Industrial Technology Department, Industrial Engineering and Robotics Faculty, National University of Science and Technology Politehnica Bucharest, Splaiul Independenței 313, 060042 Bucharest, Romania; clara.soare@upb.ro; 2 Mechanical Engineering Department, Las Palmas de Gran Canaria University, 35017 Tafira, Spain; cristina.jimenez112@alu.ulpgc.es (C.J.-M.); santiago.brito@ulpgc.es (S.B.-G.); julia.mirza@ulpgc.es (J.C.M.-R.); 3 Materials Engineering and Welding Department, Transilvania University of Brasov, 500036 Brasov, Romania

**Keywords:** Ti-Mn alloys, microstructure, microhardness, corrosion

## Abstract

Titanium–manganese alloys have emerged as a promising option of β-phase titanium alloys, which have recently gained popularity thanks to their exceptional cold strength, deformability, and high specific strength. In this study, the vacuum arc melting process was used to obtain a Ti3Mn alloy, and its behavior in three physiological conditions was analyzed: at room temperature, simulated fever conditions (at 40 °C), and simulated severe infection conditions (pH = 1.2). Optical and scanning electron microscopy were employed to study the effect of Mn addition on the Ti-base alloy microstructure. It was observed the formation of fine precipitates of Mn2Ti, localized at the grain boundaries, allow for the increase in microhardness and blocked their growth. The beta phase of titanium was obtained as fine lamellae with a low level of porosity. The microhardness values were higher than those reported for cp-Ti. The electrochemical tests have shown a high resistance to corrosion in the three analyzed conditions. On the sample’s surface, there is a passive bilayer film, composed of a porous one being in contact with the physiological liquid and a compact one in contact with the bulk alloy. The results obtained suggest that Ti3Mn alloy can be a promising low-cost biomaterial for biomedical applications.

## 1. Introduction

The advancement of materials used in biomedicine has grown in the last few decades due to an increase in the elderly population, degenerative diseases, and the need for complex surgeries [1]. In this context, titanium have become one of the preferred choices in the medical field due to its excellent biocompatibility, high corrosion resistance, and appropriate mechanical properties [2,3]. Using titanium and titanium-rich alloys for orthopedic prostheses, dental implants, and biomedical devices has revolutionized modern medicine. Their role in the human body is as follows: (a) the replacement of affected parts, which includes bones (components of hip, maxillofacial devices, and knee prosthesis) and teeth (dental implants) [4]; (b) assisting an organ or tissue in its normal function as cardiac pacemakers, cochlear implants, defibrillators, and experimental heart pumps [5]; (c) repress or enhance tissue growth or proliferation, as seen in intravascular stents [6], and (d) provide temporary support of tissues as clips, adhesives, and staples for soft tissue, plates, screws, pins and fixators, wires, brackets, etc. [7].

Among the various titanium alloys, Ti-6Al-4V, as a typical α + β alloy, has become the standard in biomedical applications due to its optimal combination of mechanical strength and biocompatibility [8]. However, this alloy is not without its drawbacks, as evidenced by the potential toxicity of vanadium and aluminum, as well as its comparatively higher elastic modulus in relation to human bone. Santos et al. [9] analyzed the effect of Mn concentrations in Ti-Mn alloys, showing that for 9% Mn, a maximum tensile strength of (1046 MPa) and an elongation at a break of 4.7% are obtained, and the Young’s modulus reached 89 GPa. The higher elastic modulus can result in a phenomenon known as stress shielding, which has the capacity to hinder the successful integration of the implant with the surrounding bone tissue.

Although the Ti6Al4V alloy has good mechanical properties and is widely used, it has been found that the high concentration of Al and V can cause the release of ions during the use of implants, which can cause health problems [10]. TiNi (nitinol) is ideal for use in vascular stents and minimally invasive surgeries because it can revert to its natural form. However, it can release nickel, which can cause allergies, kidney problems, and cardiovascular issues [11].

To overcome these limitations, Al-free, V-free and Ni-free with lower elastic modulus, and new titanium alloys with alternative biocompatible beta-stabilizer elements such as Nb, Ta, Mo, Zr and Si, have been developed [12,13]. β-phase titanium alloys have recently gained popularity thanks to their exceptional cold strength, deformability, and high specific strength [14]. However, the majority of β-type Ti alloys are composed of high-cost rare earth elements [15]. Ti-xMn (titanium–manganese) alloys have emerged as a promising option, as manganese is abundant in nature; therefore, it is cheap [16] and has a lower toxicity compared with other elements [17]. Santos et al. [9] analyzed the toxicity and cytotoxicity of Ti-Mn alloys through immersion tests in simulated body fluids (SBF) and cell viability tests. They find that the Ti-Mn alloy behaves similarly to pure Ti in terms of toxicity and the release of metal ions. Mn is an indispensable element in the human body and promotes the cellular proliferation of human osteoblasts [9,18]. Based on the results obtained from biocompatibility and cytotoxicity tests performed on TiMn alloys, with Mn concentrations from 2 to 12 wt.%, Zhang et al. [19] observed that below 8 wt.% Mn, the effects on cell proliferation and the cytotoxicity of these alloys are negligible.

Manganese (Mn) is one of the fourteen essential trace elements confirmed by the World Health Organization (WHO). It plays an important role in maintaining a normal metabolism [20,21]. It can improve the mechanical properties such as hardness and ductility [22] and corrosion resistance of the material [23] and provides important reinforcement and resistance against burns [24]. These new alloys seek to offer a better balance between biocompatibility, mechanical strength, and compatibility with bone tissue, opening new possibilities in the design of implants and advanced medical devices [25,26].

Ti-Mn-Cu alloys obtained by powder metallurgy exhibited better mechanical properties due to the formation of two-phase microstructures, simultaneously with an increased antibacterial capacity, reducing porosity and the proportion of embrittlement phases [27]. It has been found that the addition of Mn to titanium allows it to stabilize its beta phase, promoting the formation of a fine lamellar structure [28]. Thus, during the solidification process of the titanium alloy, Mn tends to accumulate at the β grain boundaries, causing the nucleation free energy of the β phase to increase and the nucleation free energy of the α phase to decrease, due to the precipitation of the Mn_2_Ti phase.

Studies on titanium alloys with up to 13% Mn have shown that hardness (308–294 HV) and tensile strength (1162–938 MPa) can be increased. Good results in terms of mechanical properties were obtained for the Ti-9Mn alloy, which had both an increased ultimate tensile strength (1046 MPa) and an acceptable elongation at the break (4.7%), under conditions of an elastic modulus of 89 GPa. However, it was observed that, with increasing Mn content, the values of these characteristics start to decrease due to the pore’s appearance and the formation of titanium carbides during the alloying process [28]. Despite its potential, a deeper understanding of how Mn affects the behavior of titanium alloys under real physiological conditions is still needed. This study evaluates the performance of the alloy in the Ringer’s solution under various conditions that simulate different physiological scenarios, such as the simulation of fever states and acidic environments associated with inflammation. The choice of testing conditions is original and aims to simulate as faithfully as possible the change in tissue acidity under fever conditions. The goal of this research is to promote the development of safer, more effective, and more affordable titanium-based materials for biomedical use.

## 2. Materials and Methods

### 2.1. Material Preparation

The study alloy was manufactured National University of Science and Technology Politehnica Bucharest using Vacuum Arc Remelting (VAR) equipment MRF ABJ 900 of Materials Research Furnaces, LLC (Allenstown, NH, USA) as ingots, having about 45 g each, using trademark Ti6Al4V alloy and Mn grains (purity of 99.2%), and six remelting operations were performed to achieve homogeneity of the product [19,28]. When designing the Ti-Mn alloy recipe, the Ti6Al4V alloy was used as a base, and its chemical composition was modified by adding Mn. Therefore, small amounts of Al and V are still found in the chemical composition of the new Ti-Mn alloy. The final chemical composition of the studied alloy is 96.2% Ti, 3.0% Mn, 0.6% Al, and 0.2% V. Quenching from 1050 °C in water was performed, then samples were cut and embedded in epoxy resin. The specimens were polished using a progressive carbide grinding process with carbide sheets of increasing grit size. Mirror polishing clothes containing a 0.3 μm alumina suspension were used, in accordance with ASTM E3-11 (2017) [29].

### 2.2. Microstructural Characterization

The microstructure of the sample was examined by taking micrographs of its surfaces using an Axio Vert.A1 MAT ZEISS inverted microscope (Jena, Germany) at different magnifications. For about 5–8 s, Ti3Mn was immersed in a solution of Kroll’s reagent, and the surface exposed to the reagent was then analyzed. Also, to identify the details of the microstructure, another metallographic etching reagent with the following composition was used: 10% HF, 30% HNO_3_, and 50 mL of deionized water. High-resolution X-ray diffraction measurements were conducted at the ID31 beamline of the European Synchrotron Radiation Facility (ESRF). The sample was loaded into cylindrical slots (with a thickness of approximately 1 mm) and measured using transmission geometry with an incident X-ray energy of 75.051 keV (with a wavelength of 0.16520 Å). The measured intensities were collected using a Pilatus CdTe 2M detector of DECTRIS Ltd. (Baden-Dättwil, Switzerland), with a sample-to-detector distance of approximately 1.5 m for high-resolution measurements.

### 2.3. Microhardness and Nanoindentation Tests

Vickers microhardness measurements were performed in accordance with ASTM E384-22 [30], using the microhardness tester Future Tech FM-810 of Future-Tech Corp. (Kawasaki, Japan), with loads of 5, 25, and 50 gf (HV 0.005, HV 0.025, and HV 0.05, respectively). Ten indentations were made on the surface of the sample for each load, with a minimum spacing of 500 µm (center-to-center) to avoid interaction between indentations. The indenter dwell time was 15 s. The microhardness values were automatically calculated from the recorded diagonals and plotted graphically as a function of the number of indentations.

To determine the elastic modulus of the Ti3Mn alloy and compare it with that of Ti6Al4V, a Fisherscope H100 ultramicrodurometer (Helmut Fischer GmbH, Sindelfingen, Germany) was used in accordance with VDI/VDE 2616 Blatt 1 (2012-08). Fifteen indentations have been performed on each sample; the final load has been 1000 mN in all cases, which has been applied gradually in 25 steps, with a rest time of 1 s between two load levels.

### 2.4. Electrochemical Tests

The electrochemical tests use three electrodes: the working electrode (the material being tested), the reference electrode (Saturated Calomel Electrode, SCE), and the counter electrode (platinum electrode), according to ISO 10271:2020 [31]. The sample was immersed in Grifols Ringer’s solution in three different conditions inside the electrochemical cell: at room temperature, at 40 °C, and with the pH acidified with lactic acid to 1.2. To perform the tests, the area of each sample under investigation was measured. Each test was repeated 3 times, and the standard deviation of the main parameters was mentioned. Using a potentiostat-galvanostat, three methods were used in the following order to study the corrosion behavior of the alloys: E_corr_ vs. Time, Linear Polarization, and Electrochemical Impedance Spectroscopy.

#### 2.4.1. E_corr_ vs. Time (EVT)

Version 9.5 of the EC-Lab software “E_corr_ vs. Time” technique was applied to evaluate each sample’s one-hour corrosion potential. Following the processing stage, the data was presented as a graph illustrating the potential against time. The potential was recorded continuously for 1 h, with automatic acquisition every 300 s or in the event of variations greater than 200 mV. These conditions stabilized the corrosion potential as much as possible.

#### 2.4.2. Linear Polarization (LP)

To conduct these tests, the “Linear Polarization” technique was selected, the sample surface area was entered, and the 5 min duration was added. The preliminary scanning revealed a 0.166 mV/s time-variation relationship spanning from −25 mV to 25 mV in relation to the open circuit potential (OCP), with data collected at an interval of 0.50 s. After displaying these curves, the polarization resistance (Rp) was obtain applying “Rp Fit” analysis, and the corrosion rate (CR) was estimated.

#### 2.4.3. Potential Electrochemical Impedance Spectroscopy (PEIS)

For the impedance measurement, the selected method was “Potential Electrochemical Impedance Spectroscopy”. This measurement was performed at thirteen potentials for each condition. The maximum and lowest potential values were set to ±1.2 V against the reference electrode (SCE) in Ringer’s solution, respectively, in accordance with ISO 16773-1-4:2016 [32]. The data was presented using Nyquist and Bode diagrams, and an equivalent circuit (EC) was utilized to fit experimental values.

## 3. Results and Discussion

### 3.1. Microstructural Investigation

**Figure 1:**
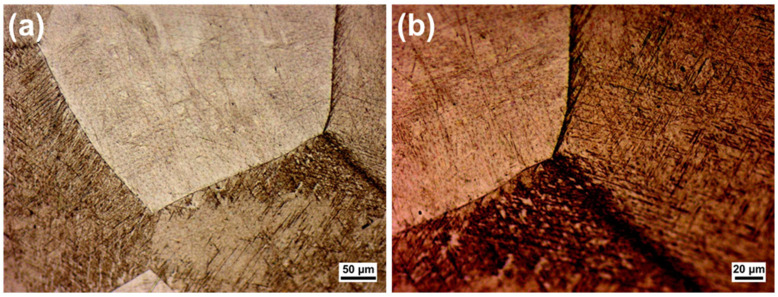
Optical microstructure of the sample after Kroll etching at (**a**) ×10 and (**b**) ×20 magnifications.

The optical microstructure of the Ti-Mn alloy specimen following chemical etching is presented in the Figure 1 at various magnifications. The image reveals equiaxed grains with well-defined boundaries, which is typical of the mixture of the α- and β-phase of titanium [23]. The slightly darkened grain boundaries suggest the presence of minor amounts of β-phase at the grain boundaries. It is known that beta-phase stabilization in titanium alloys has a huge impact on mechanical properties because it provides flexibility in the material design, offering a wider range of processing combinations of physicochemical and mechanical properties than any other class of Ti alloys. For this reason, in our alloy, the concentration of the α-stabilizer element (i.e., Al) was kept lower (0.6 wt.%) and the β-stabilizer element, like Mn, has been added (3 wt.%) [24].

**Figure 2:**
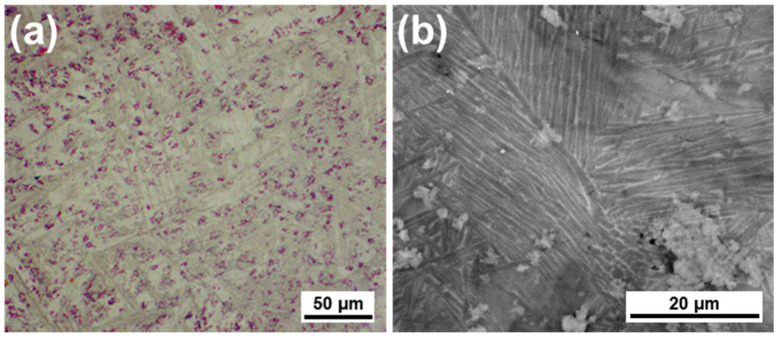
(**a**) Optical and (**b**) SEM microstructure of Ti-3Mn alloy, enhancing Mn-rich compounds.

For an increase in the β-phase proportion, quenching from 1050 °C in water has been performed. Using an etching reagent (10% HF + 30% HNO_3_ + 50 mL deionized water), the microstructure of the Ti-3Mn alloy has been observed at high magnification (Figure 2a). A stabilized acicular α/α′ in an α + β microstructure was observed in this microstructure, having uniform equiaxial grains. SEM microscopy (Figure 2b) reveals the formation of a fine acicular network of beta-titanium and the intergranular precipitation of the Mn-rich compounds, which can limit the grain boundary displacement and stabilize the microstructure.

**Figure 3:**
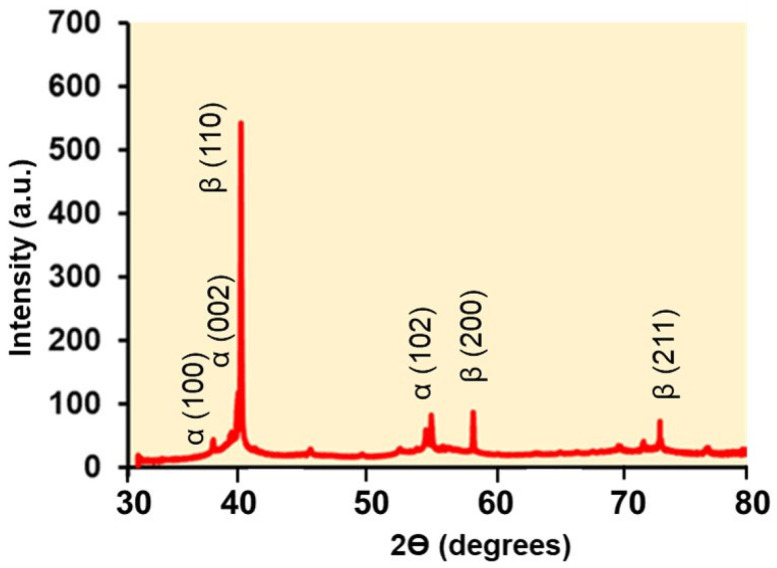
X-ray diffraction (XRD) pattern of the studied alloy.

Figure 3 shows the XRD profiles of Ti-3Mn, with the diffraction peaks of the β-Ti planes (110), (200), and (211). Although, in small amounts, the presence of Al and V also favors the appearance of the α-Ti phase, with diffraction peaks of planes (002), (100), and (102).

### 3.2. Microhardness Test

**Figure 4:**
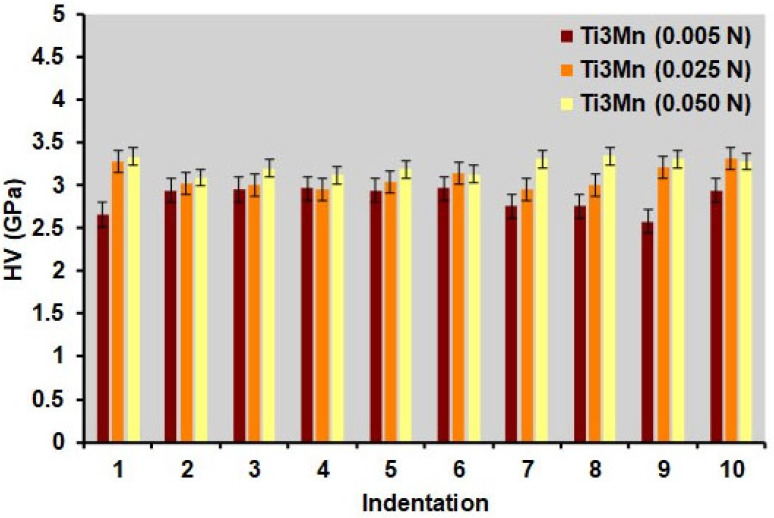
Microhardness results for the ten indentations on the test specimen (error bars with confidence interval of 95%, under loadings of 0.005, 0.025, and 0.050 N).

Microhardness measurements were performed on the experimental alloy using loads of HV 0.005, HV 0.025, and HV 0.05, following the ASTM E384 standard. The mean, median, standard deviation (SD), maximum, and minimum of the Vickers hardness and indentation depths for each set of ten measurements are shown in Figure 4 and Table 1.

The data show minimal variation between the three applied loads, with the maximum hardness value recorded at 0.05 N and the minimum at 0.005 N. The standard deviation decreases slightly at higher loads because greater penetration reduces the effect of surface heterogeneities. Indentation depths increased from 1.14 × 10^−6^ m to 3.39 × 10^−6^ m as the load increased. The dispersion of microhardness values is greater at 5 gf, while at 50 gf, the distributions are narrow, indicating greater homogeneity in the material’s response under higher loads. The median and mean coincide approximately only when applying 50 gf, reflecting symmetrical distributions without significant outliers, while for lower loads, the mean and median are further apart.

In the bar chart, for 95% confidence intervals (CI), the amplitude of the error bars decreases progressively from ±4.58 × 10^−2^ GPa at 5 gf to 3.03 × 10^−2^ GPa at 50 gf. This reduction reflects a greater measurement accuracy under higher loads, in line with the lower dispersion. The overlap of the confidence intervals confirms that the differences between the three loads are not statistically significant, although there is a slight tendency for the average hardness to increase with the applied load.

Microhardness measurements have been taken across the largeof the sample. The measured hardness increases with the load and the dispersion decreases. For small loads, indentation can be performed on a single phase or grain (favorable orientation, Mn micro-segregation, pores, etc.), giving lower and more dispersed values. As the load increases, the indentation size increases, averaging a larger volume that can contain several grains or phases, and, therefore, the measurement converges to the volume hardness, which is higher and more stable.

The elastic modulus obtained for Ti3Mn is 91.1 ± 0.7 GPa, lower than that of the Ti6Al4V alloy, which is 121.3 ± 1.2 GPa. This means that the Ti3Mn alloy is less rigid than Ti6Al4V, thereby reducing the stress-shielding effect. The Ti3Mn alloy could offer an optimal compromise between mechanical strength and bone compatibility for implants.

### 3.3. Electrochemical Tests

Lactic acid buildup is a process that occurs during vigorous activity, resulting in an acute burning sensation as the muscles generate energy in the absence of adequate oxygen.

Mabilleau et al. [33] reported that lactic acid compromises the integrity of passive films, thereby diminishing the re-passivation of pure Ti implants.

Furthermore, it was shown [34] that lactic acid adversely affects the corrosion resistance of the alloys; at a lactic acid concentration of 0.075 wt% in Hank’s solution (pH = 3), the corrosion current density increases, and the impedance of the passive film is approximately two-thirds of that observed in Hank’s solution at pH 7.

#### 3.3.1. Corrosion Potential

**Figure 5:**
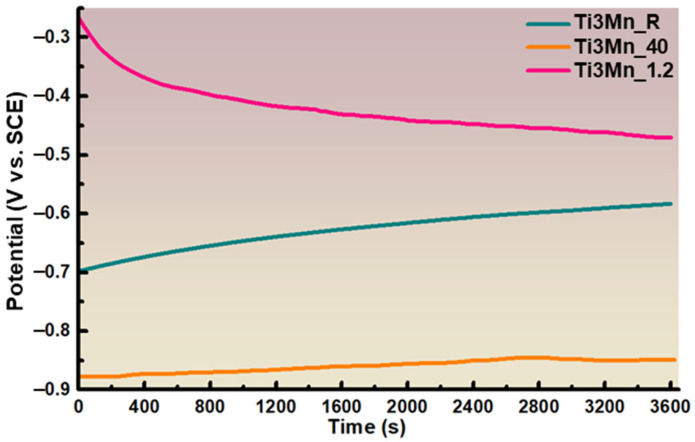
Corrosion potential curves for the sample after 1 h immersion time in Ringer’s solution at room temperature, at 40 °C, and with the pH acidified to 1.2.

As can be seen in Figure 5, the corrosion potential versus time curves of the studied sample immersed in Ringer’s solution were analyzed after a one-hour testing period under three different conditions: at room temperature (Ti3Mn_R), at 40 °C (Ti3Mn_40), and with the pH acidified to 1.2 (Ti3Mn_1.2). Under these circumstances, the potential—known as the OCP—indicates the sample’s tendency to resist corrosion. Figure 5 shows that the initial potential data of the sample immersed in Ringer’s solution at room temperature of −0.69 V and at 40 °C of −0.88 V are lower than those of the sample at pH 1.2 with −0.27 V. According to the data collected during the first 300 s of immersion, there was a drop in potential at a pH of 1.2 (−0.35 V). However, after one hour of immersion, the potential of the sample at room temperature and at 40 °C tended to increase to −0.58 V and −0.85 V, respectively, showing a tendency to passivation, but at pH 1.2, the potential value decreases to −0.47 V, and, therefore, the sample tends to corrode.

The constant movement of the potential towards noble values for the three studied conditions suggests that the passive layer underwent modifications during the test, becoming more protective.

#### 3.3.2. Polarization Resistance and Corrosion Rate

**Figure 6:**
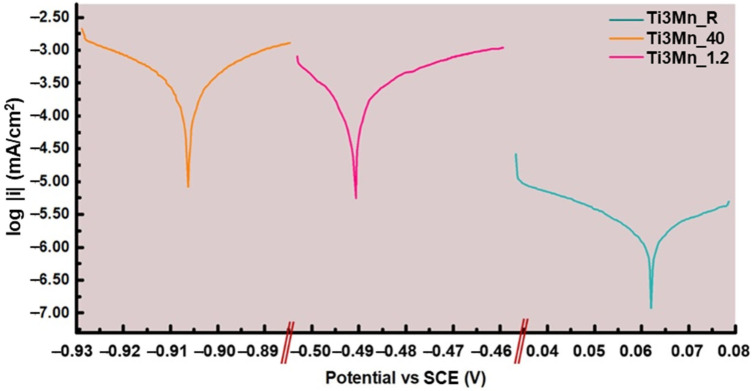
Polarization curves of the sample in Ringer’s solution at room temperature, at 40 °C, and with the pH acidified to 1.2.

Figure 6 and Table 2 illustrate the results of the linear polarization technique used to obtain the polarization resistance and estimate the corrosion rate of the alloy in the three media, presented on a semi-logarithmic scale of the actual data.

As can be seen in Table 2, the sample at room temperature has higher values of potential and lower values of the corrosion current (E_corr_ and *I_corr_*, respectively) in the Ringer’s solution at room temperature, which indicate a lower degree of oxidation of the alloy compared with the other conditions. The curve was examined against the reference potential over a full range of ±25 mV to determine the Tafel slopes (*β_c_* and *β_a_*). In this case, the analysis revealed a tendency of the study sample immersed in Ringer’s solution at room temperature to undergo the formation of a passive layer on its surface.

The equivalent weight was determined for the study sample in different conditions using the following formula, as outlined in the standard ASTM G102-23 [35]:

(1) $$EW=\frac{1}{\sum \frac{n_{i}f_{i}}{W_{i}}}$$

In this equation, “*W_i_*” represents the atomic weight of the *i*th element in the alloy, “*n_i_*” indicates its valence, and “*f_i_*” denotes its mass fraction in the alloy. As shown in Table 2, the Tafel curve parameters, the polarization resistance (*R_p_*), and the corrosion rate (CR) estimated for the tested sample are presented.

Once we have obtained the values of the polarization resistance for Ti3Mn in the three conditions using “Rp Fit”, it is essential to calculate the corrosion current (A), following the Stern–Geary equation:

(2) $$I_{corr}=\frac{\beta_{a}\cdot \beta_{c}}{R_{p}\cdot 2.3\cdot (\beta_{a}+\beta_{c})}$$

In this case, Ti3Mn immersed at room temperature presents the best Rp results of 1.06 × 10^6^ Ohm.

The calculation of these parameters is achieved through the the constant (*K*) that determines the corrosion rate units (3.27 × 10^−3^ mm·s·g/μ·cm·yr), *i_corr_* is the corrosion current density (in μA/cm^2^), and *EW* is the equivalent (g/eq) and the density *ρ* (g/cm^3^) [35].

(3) $$CR=\frac{K\cdot i_{corr}\cdot EW}{\rho }$$

A minimum *CR* of 9.00 × 10^−5^ mm·year^−1^ (3.54 × 10^−3^ mpy) was achieved at room temperature, whereas Ti-6Al-4V and cp-Ti exhibited a higher *CR* of approximately 10^−4^ mm·year^−1^ under similar conditions [36].

#### 3.3.3. Electrochemical Impedance Spectroscopy

**Figure 7:**
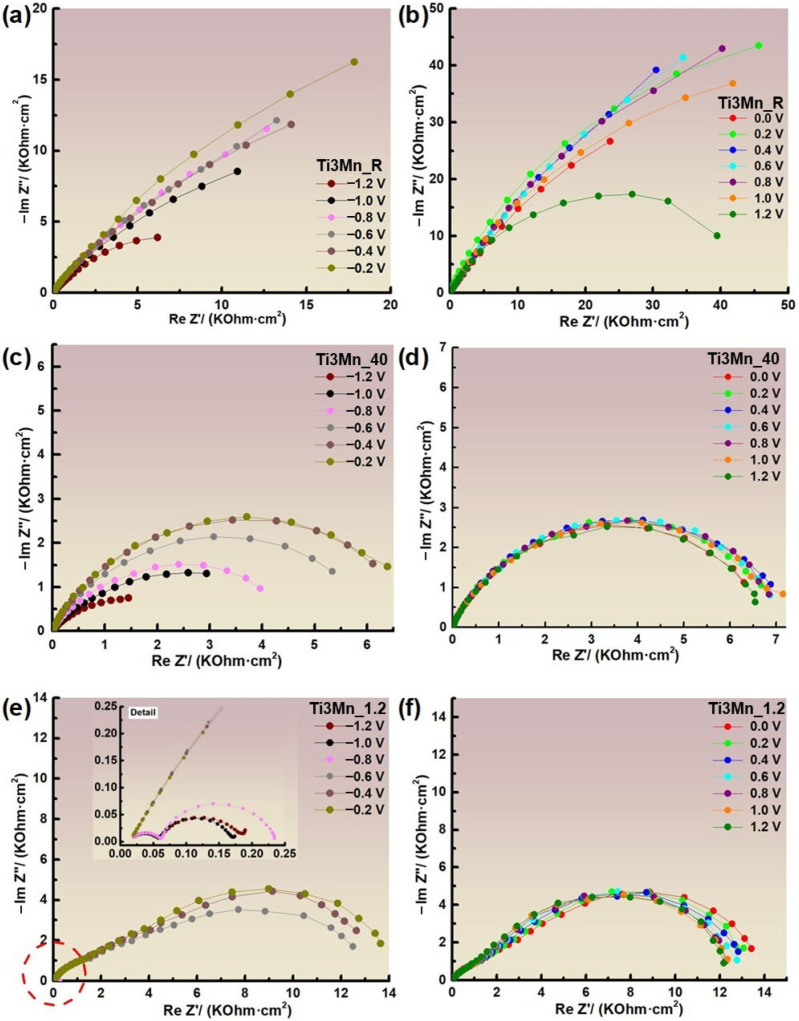
Nyquist diagrams of the sample immersed in Ringer’s solution at room temperature, at 40 °C, and at pH 1.2, from −1.2 to −0.2 V (**a**,**c**,**e**) and from 0.0 to 1.2 V (**b**,**d**,**f**), respectively.

Nyquist plots obtained for the thirteen applied potentials (±1.2 V vs. SCE with 200 mV step) at room temperature, pH 1.2, and at 40 °C, from high to low frequencies (200 KHz–100 mHz), are shown in Figure 7. In the diagrams, the imaginary impedance is plotted against the real impedance.

Analyzing the Nyquist plot at room temperature in Figure 7a,b and at 40 °C in Figure 7c,d, the impedance values tend to arc more (the total resistance is higher) when more positive potentials are applied, also showing a more capacitive behavior, except for the 1.2 V potential, which shows lower impedance values. In Ringer’s solution with pH 1.2, in Figure 7e, the formation of two arcs can be observed when applying potentials −1.2, −1.0, and −0.8 V, with a greater magnitude of −0.8 V and with very low impedance values. However, when more positive potentials are applied, a single arc is formed with much higher impedance values. Looking at Figure 7f, starting at 0 V, the arcs that form decrease as more positive potentials are applied.

**Figure 8:**
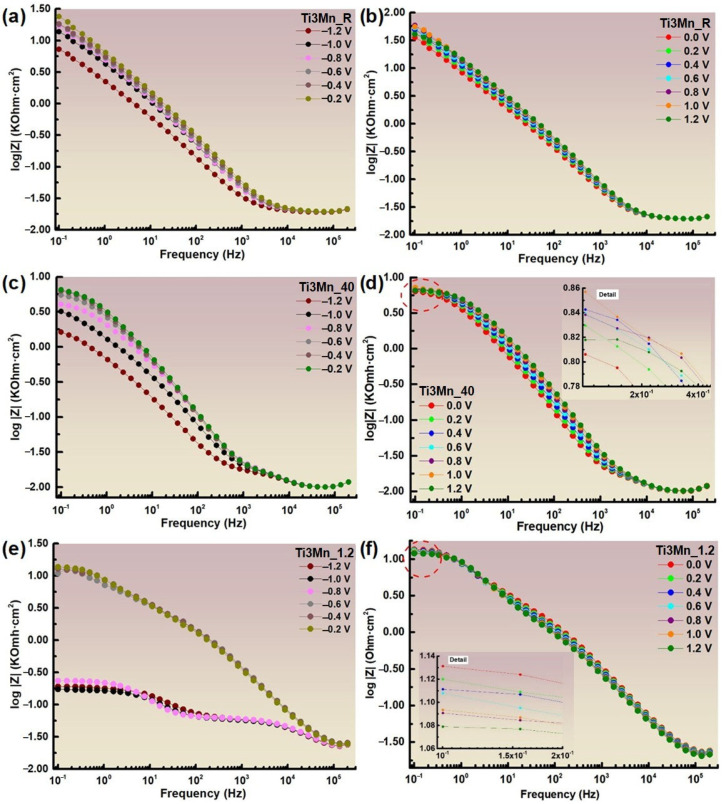
Bode impedance diagrams of the sample immersed in Ringer’s solution at room temperature, at 40 °C, and at pH 1.2, from −1.2 to −0.2 V (**a**,**c**,**e**) and from 0.0 to 1.2 V (**b**,**d**,**f**), respectively.

The lower impedance at potentials of +1.2 V is because under these conditions, the overpotential may be sufficient to initiate oxygen evolution, a reaction that involves a faster alternative charge transfer pathway, which reduces the total impedance. In addition, Mn can oxidize to more soluble states (Mn^2+^ or MnO_4_^-^), weakening the integrity of the passive layer. As a result, the layer loses its protective character and becomes more conductive, which explains the decrease in impedance at this potential. From 200 KHz to 0.1 Hz, Figure 8 displays the Bode impedance graphs for the thirteen applied potentials (±1.2 V vs. SCE) at room temperature, pH 1.2, and 40 °C.

**Figure 9:**
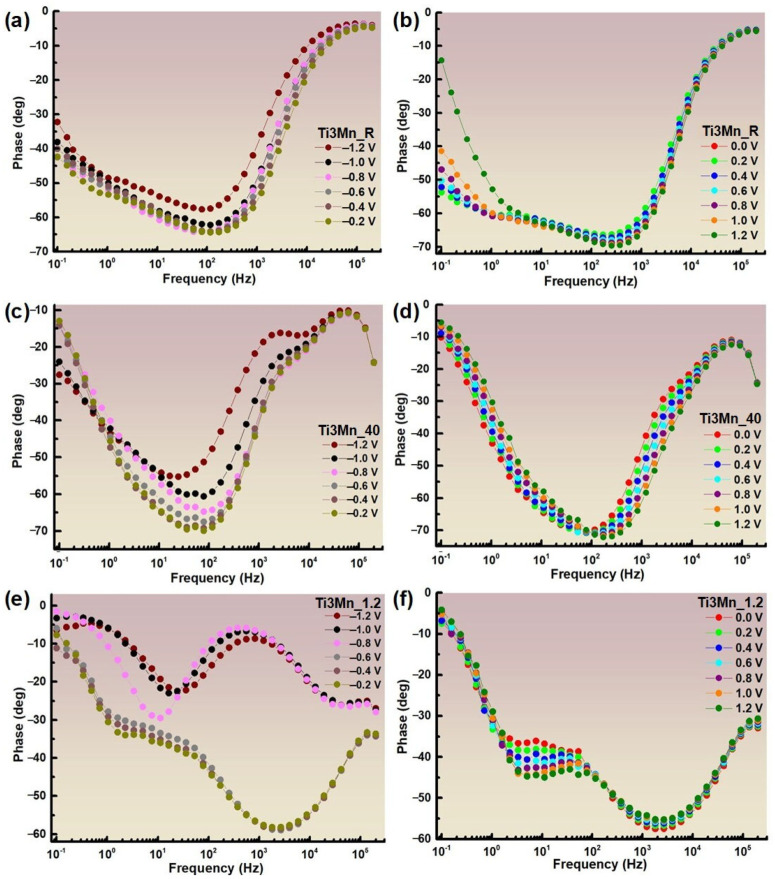
Bode-phase diagrams of the sample immersed in Ringer’s solution at room temperature, at 40 °C, and at pH 1.2, from −1.2 to −0.2 V (**a**,**c**,**e**) and from 0.0 to 1.2 V (**b**,**d**,**f**).

In the Bode-IZI plots shown in Figure 7, the impedance spectra vs. frequency is indistinguishable at high and medium frequencies. Nevertheless, a flat surface impedance is observed at a low frequency of 0.1 Hz, which is the result of the sum of the surface layer resistance, electrolyte resistance, and transfer resistance. This is related to the inherent quality of the protective film. In Figure 8a,c,e, which reflect Ringer’s conditions at room temperature, 40 °C, and pH 1.2, respectively, the impedance curves grow as more positive potentials, e −1.2 to −0.2 V, are applied. On the other hand, in Figure 8b,d,f, there is also a tendency for the impedance to increase with a more positive potential until they reach the 1.2 V potential, where the impedance decreases, with minor exceptions. The Bode-phase diagrams for the thirteen applied potentials (±1.2 V vs. SCE) at room temperature, pH 1.2, and 40 °C are displayed in Figure 9. They range from high to low frequencies (200 KHz–0.1 Hz).

As shown in Figure 9, the Bode-phase plots show phase transitions as a function of frequency for different potentials relative to the SCE. Looking at the curves for the different potentials for the three conditions studied, for the maximum frequency, the values of the phase angle are similar from −1.2 to 1.2 V for each environment. At room temperature, the maximum phase angle increases with more positive applied potentials, in this case, −1.2 V at a frequency between 10^2^ and 10^3^ Hz. On the other hand, in Ringer’s solution at 40 °C, the phase angle also increases with the application of more positive potentials, until it reaches −0.4 V at a similar frequency between 10^2^ and 10^3^ Hz, where it begins to decrease. In Ringer’s solution at pH 1.2, the value of the maximum phase angle also increases from −1.2 to −0.4 V, at a frequency between 10^3^ and 10^4^ Hz, the values from −1.2 to −0.8 V being very low in relation to the other potentials, while from −0.2 to 1.2, it decreases slightly.

In addition, the process occurs in two or three phases as the curve transitions to lower frequencies. The obtained results confirm that, in general, the more positive potentials applied in each environment, the higher the impedance values. In turn, higher impedance and phase angle values indicate a greater corrosion resistance and, therefore, the study sample presents a higher corrosion resistance immersed in Ringer’s at room temperature, followed by Ringer’s at pH 1.2, and, finally, Ringer’s at 40 °C.

**Figure 10:**
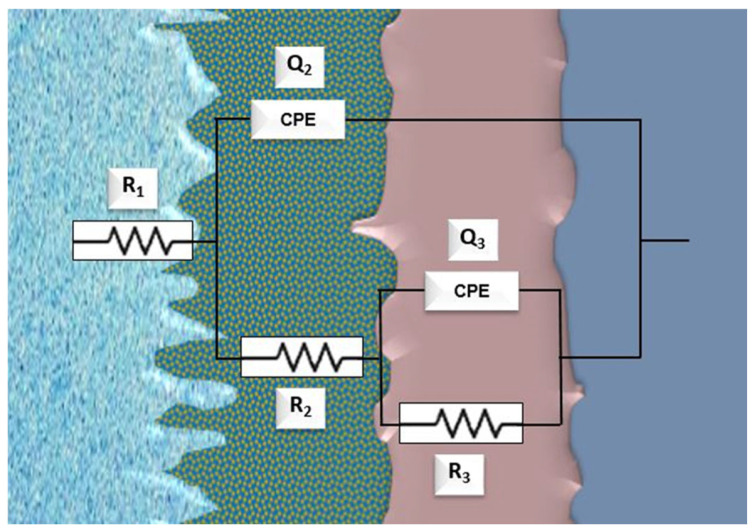
Equivalent circuit R_1_(Q_2_(R_2_(Q_3_R_3_))) applied for impedance measures at room temperature and at pH 1.2.

Figure 10 shows the applied analog circuit model R(Q(Q(R(QR)))) that best fits the experimental data in Ringer’s solutions at room temperature and pH 1.2 and shows that the sample’s surface is resistant to dissolving through the bi-layer passive film until the metallic surface is reached.

The model and the following equations represent the electrolyte resistance (*R*_1_), the constant phase elements (*Q*_2_ and *Q*_3_), as well as the resistances (*R*_2_ and *R*_3_) of the porous and compact passive films, respectively. The parameter value “*n*” of the constant phase element reproduces the resistor (*n* = 0.0), semi-infinite Warburg impedance (*n* = 0.5), and capacitor (*n* = 1.0) as a function of the applied frequency (*f*) [37]. The equivalent circuit equations are shown below:

(4) $$Z_{CPE}=\frac{1}{Y^{0}{\left(jw\right)}^{n}}$$

(5) $$Z_{parallel}={\left(\frac{1}{Z_{CPE}}+\frac{1}{Z_{R}}\right)}^{-1}$$

(6) $$Z_{Q_{3}R_{3}}={\left(\left(Y_{3}^{0}{\left(jw\right)}^{n_{3}}\right)+\frac{1}{R_{3}}\right)}^{-1}$$

(7) $$Z_{R_{2}Q_{3}R_{3}}=R_{2}+Z_{Q_{3}R_{3}}$$

(8) $$Z_{R_{2}Q_{3}R_{3}}=R_{2}+{\left(\left(Y_{3}^{0}{\left(jw\right)}^{n_{3}}\right)+\frac{1}{R_{3}}\right)}^{-1}$$

(9) $$Z_{Q_{2parallel}}={\left(\frac{1}{Z_{Q_{2}}}+\frac{1}{Z_{R_{2}Q_{3}R_{3}}}\right)}^{-1}$$

(10) $$Z_{Q_{2parallel}}={\left(Y_{2}^{0}{\left(jw\right)}^{n_{2}}+\frac{1}{Z_{R_{2}Q_{3}R_{3}}}\right)}^{-1}$$

(11) $$Z\left(f\right)=R_{1}+Z_{Q_{2parallel}}$$

(12) $$Z\left(f\right)={R_{1}+\left(Y_{2}^{0}{\left(jw\right)}^{n_{2}}+\frac{1}{R_{2}+{\left(\left(Y_{3}^{0}{\left(jw\right)}^{n_{3}}\right)+\frac{1}{R_{3}}\right)}^{-1}}\right)}^{-1}$$

The results obtained by applying the equivalent circuit (*R*_1_(*Q*_2_(*R*_2_(*Q*_3_*R*_3_)))) agreed with the experimental results. Moreover, the χ^2^ values ranged from 10^−3^ to 10^−4^, indicating that the quality of the simulation was adequate. The EIS spectra obtained for all the applied potentials, interpreted by the two-constant time equivalent circuit, indicate the existence of a bi-layer structure on the surface of the.

According to Table 3, the corrosion resistance values of the research sample (*R_p_* = *R*_2_ + *R*_3_) at room temperature and pH 1.2 tend to increase during the potential scan and reach values up to 10^5^ Ohm·cm^2^, in the case of room temperature, while at pH 1.2, they reach 10^4^ Ohm·cm^2^.

**Figure 11:**
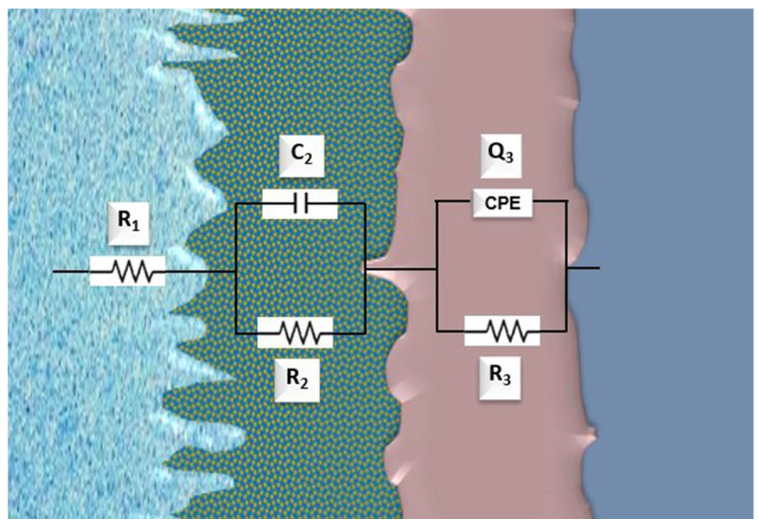
Equivalent circuit *R*_1_(*C*_2_*R*_2_) (*Q*_3_*R*_3_) applied for the impedance measures at room temperature and at 40 °C.

The outer porous film presents a lower corrosion resistance than the inner compact film, as observed by the higher values of *R*_3_ compared to those of *R*_2_. In addition, as the applied voltage becomes more positive, the sample shows higher resistance values. The thickening of the passive film is the cause of this increase in resistance as the applied potential rises to positive values. As additional positive potentials are applied, the corrected values of *Y*_2_ and *Y*_3_ decrease at room temperature, suggesting that the stability and compactness of the passive protective film have improved. The applied analog circuit model *R*(*CR*)(*QR*), which is displayed in Figure 11, best matches the experimental findings of the Ringer’s solution investigation at 40 °C. It demonstrates that the sample surface and the double passive layer (porous and compact) are resistant to dissolving until alloying is accomplished.

In this case, *R*_1_ is retained as the dissolution resistance, *C*_2_ is the capacitor, and *R*_2_ is the porous passive layer resistance of the sample surface, while *Q*_3_ is the phase constant element, and *R*_3_ is the compact passive layer resistance. Based on equivalent circuits, the circuit equations are as follows:

(13) $$Z_{C}=\frac{1}{jwC}$$

(14) $$Z_{C_{2}R_{2}}={\left(\frac{1}{Z_{C_{2}}}+\frac{1}{R_{3}}\right)}^{-1}$$

(15) $$Z_{C_{2}R_{2}}={\left(jwC_{2}+\frac{1}{R_{2}}\right)}^{-1}$$

(16) $$Z\left(f\right)=R_{1}+Z_{C_{2}R_{2}}+Z_{Q_{3}R_{3}}$$

(17) $$Z\left(f\right)=R_{1}+{\left(jwC_{2}+\frac{1}{R_{2}}\right)}^{-1}+{\left(\left(Y_{3}^{0}{\left(jw\right)}^{n_{3}}\right)+\frac{1}{R_{3}}\right)}^{-1}$$

The calculated values for this situation are presented in Table 4. The corrosion resistance values of the experimental alloy at 40 °C show a slight tendency to increase during the potential scan, reaching values up to 10^3^ Ohm·cm^2^.

As in the other two environments, the inner compact layer exhibits a higher resistance (*R*_3_) more than the outer porous film (*R*_2_), which contributes to a thickening of the passive film.

So, at ambient temperature and pH = 1.2, the best fit of the experimental data was obtained with a circuit having two CPEs, indicating that the electrochemical response is not ideally capacitive. This reflects that the passive oxide film is not homogeneous, having two distinct regions: a hydrated, porous outer layer and a compact inner layer attached to the alloy.

At a temperature of 40 °C, the system is electrochemically simplified, since one of the responses behaves ideally (it can be modeled with a capacitor C and not with a CPE), suggesting that, due to the temperature, the layer is more homogeneous at the oxide/electrolyte interface; in addition, greater ionic conductivity is observed due to the effect of the temperature.

## 4. Conclusions

The major results of this research study can be synthesized as follows:

1. The vacuum arc remelting method followed by quenching at 1050 °C and water cooling were employed for the synthesis of the Ti3Mn alloy and allowed us to obtain the required microstructure.
2. The incorporation of Mn into titanium caused the formation of fine precipitates of Mn-rich features at the grain boundaries, which can limit the grain boundary expansion. The mixture of acicular α/α′ in an α + β microstructure of titanium was obtained, having a reduced porosity, and being an optimal profile for biomedical applications.
3. The Ti3Mn alloy exhibited microhardness levels that exceeded commercially pure titanium (cp-Ti), although inferior to Ti-6Al-4V.
4. Under the three physiological conditions that were simulated (room temperature, simulated fever at 40 °C, and acidic medium pH 1.2), the alloy exhibited good corrosion resistance. A passive double-layer film was identified on the alloy surface, composed of a porous outer film in contact with the physiological medium and a compact inner one in contact with the base metal, which contributes to corrosion protection.
5. The derived findings suggest that the Ti3Mn alloy has considerable potential as a biomaterial for medical uses, attributable to its conjunction of mechanical and chemical characteristics.

## Figures and Tables

**Table 1:** Microhardness values and depth of the sample.

Essay	HV (GPa)					Depth (m)
	Mean	Median	SD	Maximum	Minimum	
0.005 N	2.85	2.94	0.14	2.96	2.58	1.14 × 10^−6^
0.025 N	3.09	3.03	0.13	3.32	2.95	2.45 × 10^−6^
0.050 N	3.23	3.24	0.10	3.35	3.09	3.3 × 10^−6^

**Table 2:** Corrosion parameters obtained for the sample.

Parameters	Room Temperature	40 °C	pH 1.2
E_corr_ ± SD(V vs. SCE)	(6.16 ± 0.23) × 10^−2^	(−9.08 ± 0.62) × 10^−1^	(−4.91 ± 0.44) × 10^−1^
*I_corr_* ± SD (A)	(5.12 ± 0.34) × 10^−9^	(4.93 ± 0.23) × 10^−7^	(1.84 ± 0.05) × 10^−7^
A (m^2^)	5.00 × 10^−5^	5.00 × 10^−5^	5.00 × 10^−5^
*EW* (Kg/eq)	1.22 × 10^−2^	1.22 × 10^−2^	1.22 × 10^−2^
*ρ* (Kg/m^3^)	4.54 × 10^3^	4.54 × 10^3^	4.54 × 10^3^
*β_c_* (V/dec)	23.10 × 10^−3^	24.50 × 10^−3^	17.30 × 10^−3^
*β_a_* (V/dec)	26.90 × 10^−3^	24.80 × 10^−3^	16.50 × 10^−3^
*R_p_* ± SD (Ohm)	(1.06 ± 0.07) × 10^6^	(1.14 ± 0.10) × 10^4^	(2.00 ± 0.05) × 10^4^
Correlation	0.9989	0.9998	0.9959
*CR* ± SD (mm·year^−1^)	(9.00 ± 0.60) × 10^−5^	(8.66 ± 0.40) × 10^−3^	(3.23 ± 0.08) × 10^−3^

**Table 3:** Equivalent electric circuit parameters for fitting the experimental EIS data at room temperature and at pH 1.2.

Potential (V)	Conditions	*Y*_2_ (S·sec^n^/cm^2^)	n_2_	*R*_2_ (Ω·cm^2^)	*Y*_3_ (S·sec^n^/cm^2^)	n_3_	*R*_3_ (Ω·cm^2^)	χ^2^
−1.2	Room Temp.	3.93 × 10^−5^	0.81	6.92 × 10^2^	1.08 × 10^−4^	0.57	1.49 × 10^4^	7.57 × 10^−4^
−1.0		1.62 × 10^−5^	0.84	4.99 × 10^2^	5.94 × 10^−5^	0.49	5.63 × 10^4^	7.39 × 10^−4^
−0.8		1.42 × 10^−5^	0.86	3.28 × 10^2^	5.17 × 10^−5^	0.47	1.19 × 10^5^	7.34 × 10^−4^
−0.6		1.48 × 10^−5^	0.85	5.22 × 10^2^	5.32 × 10^−5^	0.48	9.96 × 10^4^	7.17 × 10^−4^
−0.4		1.21 × 10^−5^	0.85	6.13 × 10^2^	4.76 × 10^−5^	0.48	1.14 × 10^5^	7.74 × 10^−4^
−0.2		1.31 × 10^−5^	0.84	1.09 × 10^3^	3.83 × 10^−5^	0.53	1.06 × 10^5^	7.87 × 10^−4^
0.0		9.95 × 10^−6^	0.86	1.05 × 10^3^	2.68 × 10^−5^	0.57	2.52 × 10^5^	7.88 × 10^−4^
0.2		7.75 × 10^−6^	0.89	7.34 × 10^2^	9.78 × 10^−6^	0.64	1.39 × 10^5^	8.40 × 10^−4^
0.4		7.18 × 10^−5^	0.90	8.28 × 10^2^	8.86 × 10^−6^	0.65	1.28 × 10^5^	8.38 × 10^−4^
0.6		6.76 × 10^−6^	0.90	8.66 × 10^2^	8.19 × 10^−6^	0.65	1.28 × 10^5^	9.05 × 10^−4^
0.8		5.84 × 10^−6^	0.88	1.55 × 10^3^	1.50 × 10^−5^	0.63	1.49 × 10^4^	7.84 × 10^−4^
1.0		5.50 × 10^−6^	0.89	1.75 × 10^3^	1.27 × 10^−5^	0.65	1.26 × 10^5^	8.06 × 10^−4^
1.2		5.30 × 10^−6^	0.89	2.01 × 10^3^	1.03 × 10^−5^	0.68	4.70 × 10^4^	8.50 × 10^−4^
−1.2	pH = 1.2	3.96 × 10^−5^	0.53	57.50	2.55 × 10^−4^	0.81	1.19 × 10^2^	3.89 × 10^−3^
−1.0		3.48 × 10^−5^	0.54	55.90	2.61 × 10^−4^	0.88	1.03 × 10^2^	3.35 × 10^−3^
−0.8		2.50 × 10^−5^	0.57	56.10	2.90 × 10^−4^	0.91	1.63 × 10^2^	3.39 × 10^−3^
−0.6		5.07 × 10^−6^	0.71	2.93 × 10^3^	3.50 × 10^−5^	0.62	1.17 × 10^4^	5.83 × 10^−4^
−0.4		5.71 × 10^−6^	0.70	3.40 × 10^3^	2.52 × 10^−5^	0.71	1.03 × 10^4^	6.89 × 10^−3^
−0.2		6.24 × 10^−6^	0.70	3.30 × 10^3^	2.09 × 10^−5^	0.72	1.19 × 10^4^	5.01 × 10^−3^
0.0		7.13 × 10^−6^	0.69	3.23 × 10^3^	1.81 × 10^−5^	0.77	1.15 × 10^4^	4.67 × 10^−3^
0.2		8.01 × 10^−6^	0.69	3.05 × 10^3^	1.57 × 10^−5^	0.80	1.12 × 10^4^	4.74 × 10^−3^
0.4		8.92 × 10^−6^	0.68	2.96 × 10^3^	1.30 × 10^−5^	0.83	1.09 × 10^4^	4.52 × 10^−3^
0.6		9.61 × 10^−6^	0.68	2.90 × 10^3^	1.06 × 10^−5^	0.86	1.06 × 10^4^	4.42 × 10^−3^
0.8		1.01 × 10^−5^	0.67	2.78 × 10^3^	9.47 × 10^−6^	0.88	1.05 × 10^4^	4.28 × 10^−3^
1.0		1.03 × 10^−5^	0.67	2.70 × 10^3^	8.24 × 10^−6^	0.89	1.03 × 10^4^	4.30 × 10^−3^
1.2		1.06 × 10^−6^	0.67	2.66 × 10^3^	7.34 × 10^−6^	0.89	1.02 × 10^4^	4.41 × 10^−3^

**Table 4:** Equivalent electric circuit values that fit the experimental EIS data at 40 °C.

Potential (V)	*C*_1_ (F/cm^2^)	*R*_1_ (Ω·cm^2^)	*Y*_2_ (S·sec^n^/cm^2^)	*n*_2_	*R*_2_ (Ω·cm^2^)	χ^2^
−1.2	2.99 × 10^−8^	10.80	3.66 × 10^−4^	0.66	2.23 × 10^3^	9.48 × 10^−3^
−1.0	2.88 × 10^−8^	10.23	1.58 × 10^−4^	0.70	3.67 × 10^3^	9.70 × 10^−3^
−0.8	2.83 × 10^−8^	10.30	8.45 × 10^−5^	0.74	4.32 × 10^3^	7.17 × 10^−3^
−0.6	2.88 × 10^−8^	10.50	6.72 × 10^−5^	0.77	5.88 × 10^3^	6.80 × 10^−3^
−0.4	2.93 × 10^−8^	10.70	6.07 × 10^−5^	0.79	6.77 × 10^3^	7.38 × 10^−3^
−0.2	2.93 × 10^−8^	10.71	5.35 × 10^−5^	0.80	6.79 × 10^3^	7.05 × 10^−3^
0.0	2.89 × 10^−8^	10.65	4.44 × 10^−5^	0.81	6.66 × 10^3^	6.09 × 10^−3^
0.2	2.88 × 10^−8^	10.42	3.76 × 10^−5^	0.81	6.88 × 10^3^	5.34 × 10^−3^
0.4	2.84 × 10^−8^	10.30	3.35 × 10^−5^	0.81	7.03 × 10^3^	4.86 × 10^−3^
0.6	2.81 × 10^−8^	10.26	3.00 × 10^−5^	0.81	6.95 × 10^3^	4.96 × 10^−3^
0.8	2.77 × 10^−8^	10.14	2.69 × 10^−5^	0.81	6.91 × 10^3^	5.03 × 10^−3^
1.0	2.74 × 10^−8^	9.97	2.38 × 10^−5^	0.82	6.80 × 10^3^	5.29 × 10^−3^
1.2	2.73 × 10^−8^	9.93	2.13 × 10^−5^	0.82	6.48 × 10^3^	5.53 × 10^−3^

## Data Availability

The original contributions presented in this study are included in the article. Further inquiries can be directed to the corresponding author.
